# Mistletoe extract Fraxini inhibits the proliferation of liver cancer by down-regulating c-Myc expression

**DOI:** 10.1038/s41598-019-41444-2

**Published:** 2019-04-23

**Authors:** Peiying Yang, Yan Jiang, Yong Pan, Xiaoping Ding, Patrea Rhea, Jibin Ding, David H. Hawke, Dean Felsher, Goutham Narla, Zhimin Lu, Richard T. Lee

**Affiliations:** 1 Departments of Palliative, Rehabilitation, and Integrative Medicine, The University of Texas MD Anderson Cancer Center, Houston, Texas USA; 2 Hubei Institute for Food and Drug Control, Wuhan, Hubei P.R. China; 3 Department of System Biology, The University of Texas MD Anderson Cancer Center, Houston, Texas USA; 4 Department of Medicine and Pathology, Stanford University School of Medicine, San Francisco, California, USA; 5 Division of Genetic Medicine, Department of Internal Medicine, University of Michigan, Ann Arbor, Michigan USA; 6 Department of Neuro-Oncology, The University of Texas MD Anderson Cancer Center, Houston, Texas USA; 7 Department of Medicine, Case Western Reserve University & University Hospitals, Cleveland, Ohio, USA

**Keywords:** Cancer, Drug development

## Abstract

Mistletoe (Viscum album) is a type of parasitic plant reported to have anticancer activity including in hepatocellular carcinoma (HCC). However, the mechanism of mistletoe’s anticancer activity, and its effectiveness in treating HCC are not fully understood. We report here that mistletoe extracts, including Fraxini (grown on ash trees) and Iscador Q and M (grown on oak and maple trees), exert strong antiproliferative activity in Hep3B cells, with median inhibitory concentrations (IC_50_) of 0.5 µg/mL, 7.49 µg/mL, and 7.51 µg/mL, respectively. Results of Reversed Phase Proteomic Array analysis (RPPA) suggests that Fraxini substantially down-regulates c-Myc expression in Hep3B cells. Fraxini-induced growth inhibition (at a concentration of 1.25 μg/ml) was less pronounced in c-Myc knockdown Hep3B cells than in control cells. Furthermore, in the Hep3B xenograft model, Fraxini-treated (8 mg/kg body weight) mice had significantly smaller tumors (34.6 ± 11.9 mm^3^) than control mice (161.6 ± 79.4 mm^3^, p < 0.036). Similarly, c-Myc protein expression was reduced in Fraxini treated Hep3B cell xenografts compared to that of control mice. The reduction of c-Myc protein levels *in vitro* Hep3B cells appears to be mediated by the ubiquitin-proteasome system. Our results suggest the importance of c-Myc in Fraxini’s antiproliferative activity, which warrants further investigation.

## Introduction

The global incidence of hepatocellular carcinoma (HCC) has steadily risen in the last two decades^1–5^. In the United States, the rising incidence and prevalence of HCC are attributable to the increased number of people living with chronic hepatitis C virus infection^5^. HCC has a poor prognosis, with overall 5-year survival rates of less than 15%^2,6^. Only 15–20% of patients are considered surgical or liver transplant candidates at diagnosis and, unfortunately, conventional chemotherapies have had limited therapeutic benefit in advanced HCC.

In the last decade, molecular characterization of HCC has identified aberrant signaling pathways and has led to the development of targeted agents for the treatment of advanced HCC. For example, sorafenib, which inhibits both the Ras/Raf/Mek/Erk pathway and angiogenic receptors, has been approved by the United States Food and Drug Administration for treating HCC. However, sorafenib provides only a 2.8-month improvement in median overall survival compared to placebo^7^. Studies using combinations of sorafenib and other antiangiogenic or targeted agents have yielded limited additional benefit^8^. Regorafenib, another antiangiogenic tyrosine kinase inhibitor, was approved for second-line therapy, but like Sorafenib it provides only modest survival benefits. Most recently, nivolumab (an immunotherapy) has been approved for advanced HCC but similar to other targeted agents, has a limited response rate of 15–20% with over 90% of patients eventually progressing on treatment^9^. Thus, effective systemic therapies that regulate the aberrant molecular pathways in HCC are needed.

Of these molecular signaling pathways, *MYC* plays a critical role in regulating the development of HCC^10–12^. *MYCC*, together with *MYCL* and *MYCN*, are the most commonly overexpressed genes in human cancer, and *MYCC* expression is highly regulated and closely linked to cell growth, apoptosis, and differentiation^12,13^. Both hepatitis B and C virus genes can potentiate c-Myc-induced tumorigenesis in transgenic mice, and the c-Myc pathway also is essential in nonalcoholic steatohepatitis-associated HCC models^14–16^, which suggests a central role for c-Myc in HCC, regardless of the etiology of disease. In humans, c-Myc is overexpressed in up to 70% of tumor tissues from patients with viral or alcohol-related HCC^17^, and c-Myc amplification has been linked to a more aggressive phenotype in HCC patients^18^. Sridharan and colleagues reported that c-Myc is one of four important factors that maintain the cancer stem cell phenotype in HCC^19,20^. The function of c-MYC makes it a highly attractive target for anti-cancer therapy. MYC itself is a challenging therapeutic target because of the paucity of targetable sites for the development of small molecule inhibitors thus far^21^. Small molecules have been developed to target the CMYC oncogene, however, to date these agents have not been approved clinically^22^. Collectively, these studies suggest that a pharmaceutically tractable c-Myc targeting approach would represent a novel treatment paradigm for HCC patients.

Complementary and alternative medicines are gaining more attention in oncology management^23,24^. Natural products from plants and animals were the source of medicinal preparations and, more recently, natural products have continued to enter clinical trials as anticancer and antimicrobial agents^25,26^. Natural products have been valuable sources for new therapeutic agents as 41% of FDA approved anticancer drugs are derived from natural compounds^27^. Mistletoe extract (ME; *Viscum album L*) can potentially spare normal cells and selectively inhibit proliferation or induce apoptosis in cancer cells. Mistletoe has been used for treating different cancers, including HCC, in Europe for over 80 years^28–33^. Different MEs are prepared from mistletoe grown on different host trees, such as fir (*Abies*), maple (*Acer*), oak (*Quercus*), apple. Of the various ME, Viscum Fraxini (Fraxini), grown on ash trees (*Fraxinus*), has the highest content of bioactive/cytotoxic mistletoe lectins (MLs) (>10 µg/ml)^32^. Intratumoral injection of Fraxini (8 mg/kg) into a human pancreatic cancer xenograft model resulted in significant tumor response with 75% of treated mice having either a partial response or complete response^34^. A phase II clinical trial reported that 20% of chemotherapy-naïve patients with advanced HCC had either complete response (13.1%) or partial response (8.1%) with limited toxicity after subcutaneous injection of Fraxini^32^. Clinical studies also have demonstrated that ME significantly reduced tumor growth and improved quality of life^35^. However, the molecular mechanisms associated with Fraxini’s anticancer activity in HCC are largely unknown.

We hypothesized that the antiproliferative effect of MEs (specifically Fraxini) in HCC cells and in xenograft models is potentially through targeting the c-Myc signaling in HCC. By understanding the mechanism behind the anticancer activity of Fraxini in liver cancer, and we hope to expand our understanding and potentially identify HCC patients that would be benefit from Fraxini derived treatment.

## Results

### Anti-proliferative effects of MEs in human HCC cells and xenograft model

**Figure 1:**
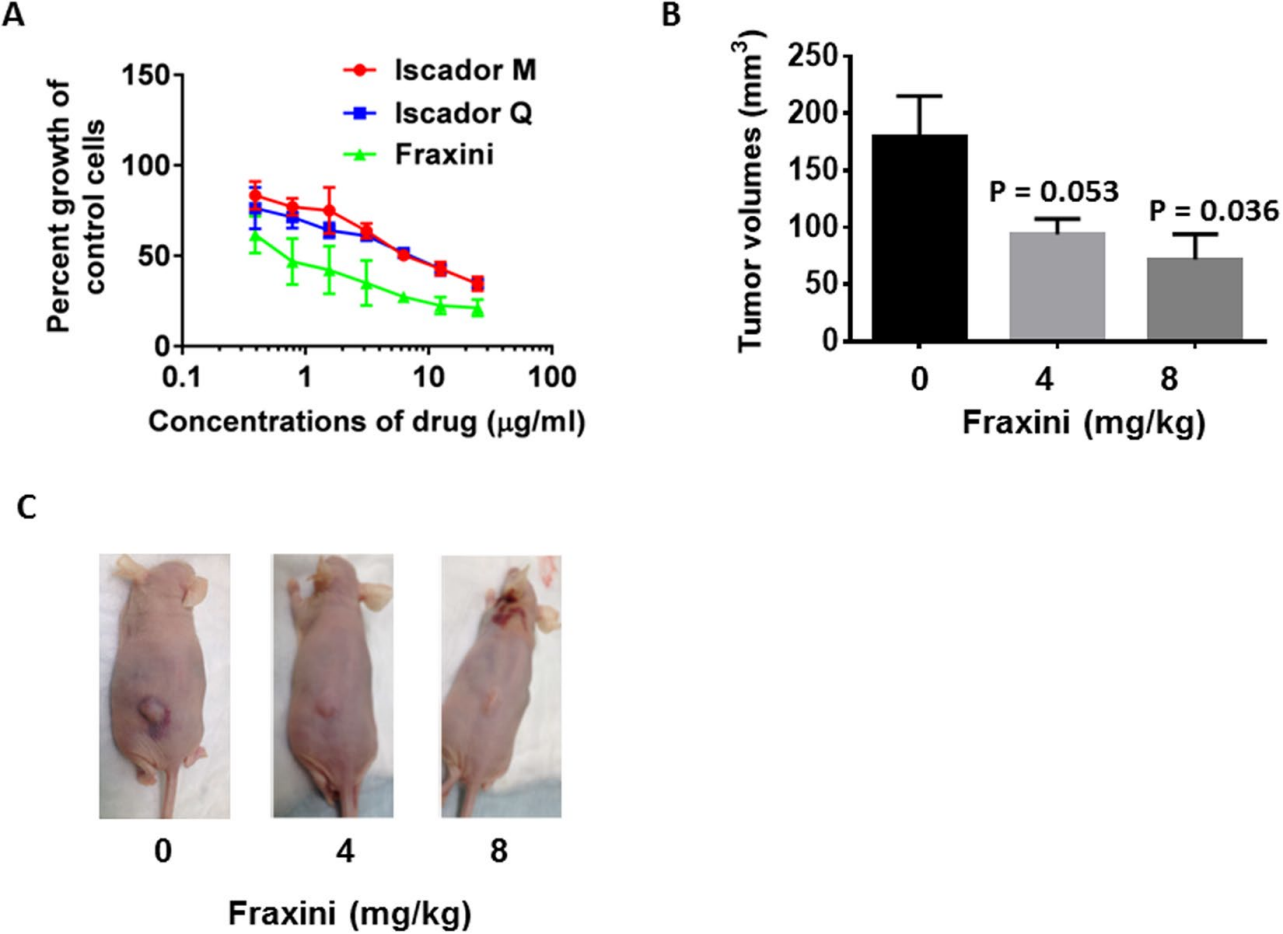
Mistletoe extracts exhibited anticancer effects in hepatocellular carcinoma Hep3B cells. The anti-proliferative effect of three mistletoe extracts in Hep3B cells (**A**). Hep3B cells were treated with different concentrations of Iscador Q (grown on oak trees), Iscador M (grown on maple trees), or Fraxini (grown on ash trees) for 72 hr. Data are mean ± SD of three separate experiments. (**B**) Fraxini reduced tumorigenesis in a Hep3B xenograft mouse model. (**C**) Photographs of tumors in representative control and Fraxini-treated mice.

To identify the sensitivity of HCC cells to ME, we first examined the effects of several MEs on the Hep3B cell line. As shown in Fig. 1A, all three MEs (Fraxini, Iscador Q, and Iscador M) exerted antiproliferative activity in Hep3B cells, with median inhibitory concentrations (IC_50_) of 0.5 µg/ml, 7.49 µg/ml, and 7.51 µg/ml, respectively. The antiproliferative effect of Fraxini was 15 times stronger than that of Iscador M and Iscador Q, respectively, in Hep3B cells. To further evaluate the efficacy of Fraxini in HCC, we conducted an antitumor efficacy study in a Hep3B cell mouse xenograft model. Fraxini treatment for 3 weeks reduced tumor volume in a dose-dependent manner. The average tumor volume in mice treated with 8 mg/kg of Fraxini (34.6 ± 11.86 mm^3^) was 61% lower than the average tumor size of the control mice (161.6 ± 79.4 mm^3^) (Fig. 1B,C) (P < 0.05).

### Induction of apoptosis by Fraxini

**Figure 2:**
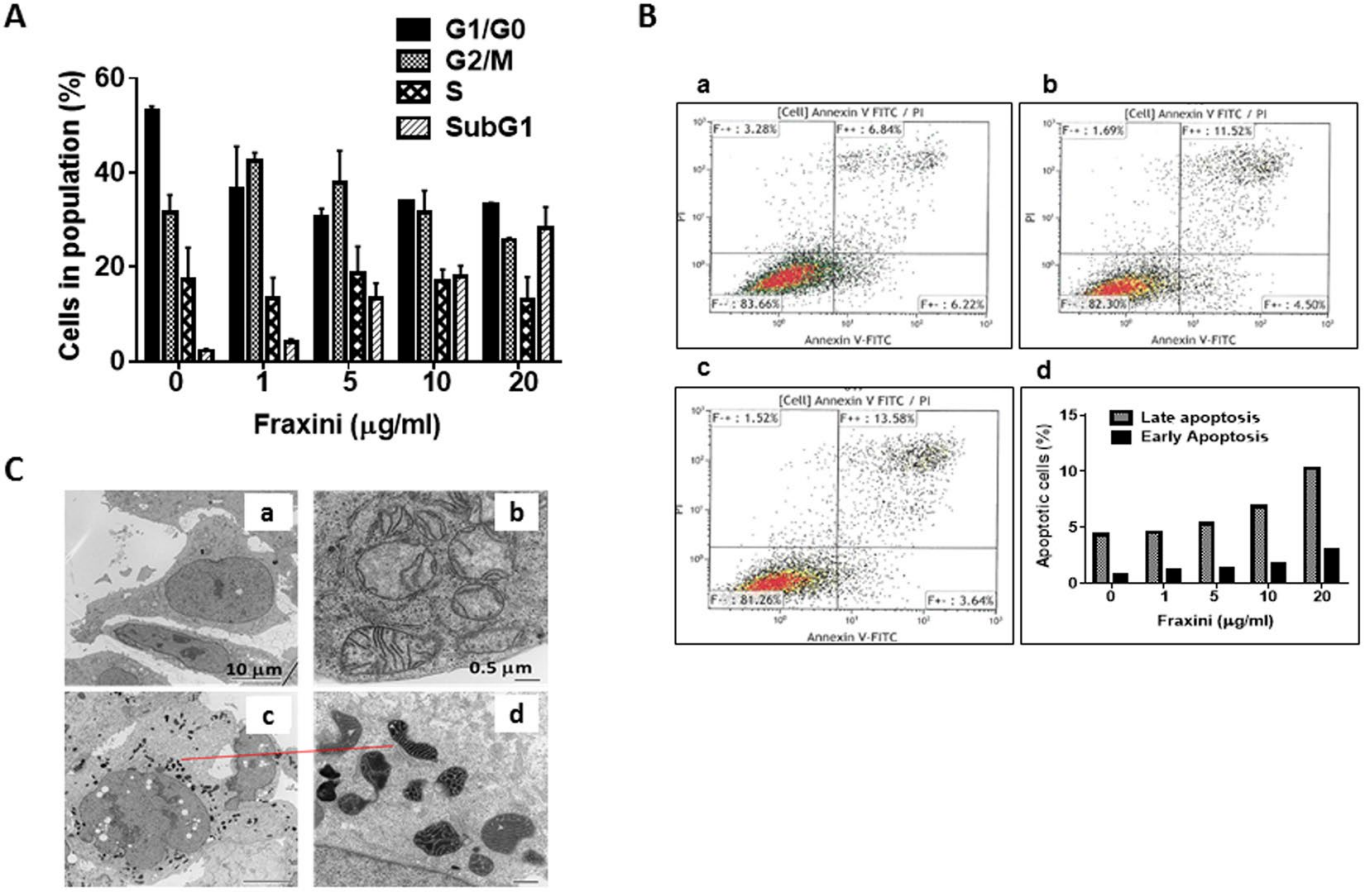
Fraxini inhibited the growth of Hep3B cells via inducing apoptosis. (**A**) Cell-cycle analysis in Hep3B cells treated with control or Fraxini (1 μg/ml–20 μg/ml); independent experiments performed in triplicate. (**B**) Flow cytometry analysis for apoptosis in Annexin V–stained Hep3B cells. a. Control; b. Fraxini (5 μg/ml); c. Fraxini (10 μg/ml); d. Quantification results for early- and late-stage apoptosis. (**C**) Compared with untreated cells (a,b), cells treated with Fraxini (10 μg/ml, c,d) had notably more vacuoles in the nuclei and had extremely condensed mitochondria in the perinuclear position.

To determine the potential mechanisms by which Fraxini inhibited the proliferation of Hep3B cells, we performed cell-cycle and apoptosis analysis. Fraxini (5 µg/ml) markedly increased the number of cells in sub-G_1_/G_0_ phase (500%) compared with the vehicle-treated samples (Fig. 2A) and the apoptotic effect of Fraxini was concentration dependent. Fraxini-treated samples had more apoptotic cells than the control samples, as quantitated by Annexin V staining (Fig. 2B).

Evaluation by transmission electron microscopy of the cellular ultrastructural changes showed that Hep3B cells treated with Fraxini (5 µg/ml) had more vacuoles in the nucleus, leading to extremely condensed mitochondria in the perinuclear position of the cells (Fig. 2C, c,d), as compared to vehicle-treated cells (Fig. 2C, a,b). This finding of mitochondrial conformational changes suggests that Fraxini-treated Hep3B cells underwent apoptosis.

### Fraxini modulates the apoptotic modulating protein and cell signaling protein, especially c-Myc

**Figure 3:**
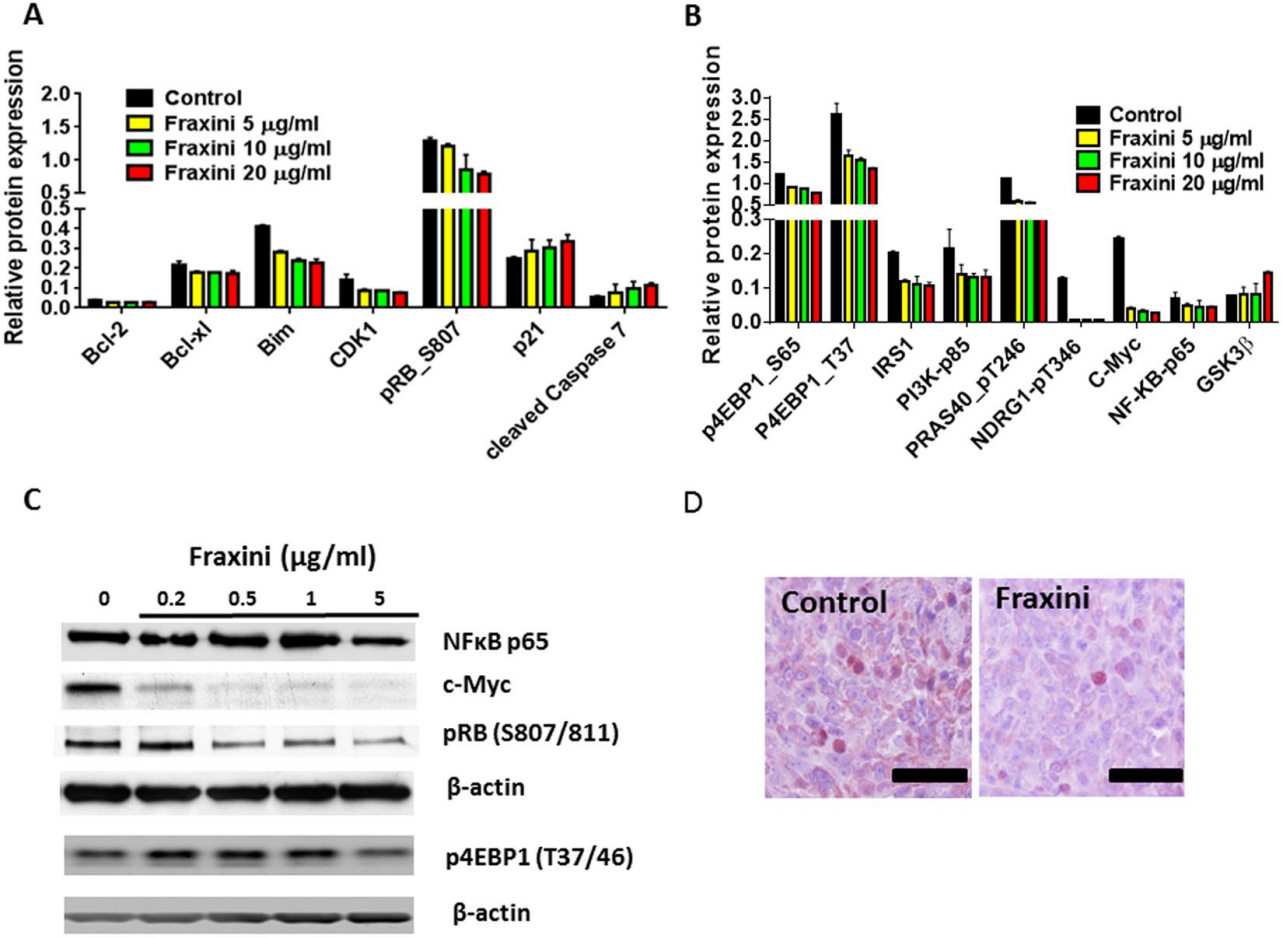
Expression of proteins that regulate apoptosis and cell signaling in Hep3B cells treated with Fraxini. (**A**) Cell cycle and apoptotic regulating protein in Fraxini treated Hep3B cells by RPPA. (**B**) Cell signaling proteins, including c-Myc were altered dramatically by Fraxini treatment. (**C**) Western blot analysis confirmed the protein expression changes observed in RPPA analysis. (**D**) Immunohistochemical staining of c-Myc protein in Hep3B tumor tissues treated with Fraxini.

To investigate the effect of Fraxini on apoptotic proteins and other signaling proteins, we performed RPPA analysis in Fraxini-treated Hep3B cells (Fig. S2A). As shown in Fig. 3A, the protein levels of Bcl-xl, Bcl2, pRb and CDK1 were reduced by Fraxini, whereas cleaved caspase 7 were elevated in a concentration-dependent manner, according to RPPA analysis results. Western blot results confirmed that, of these proteins, Fraxini increased p21 and reduced Bcl-xl levels (Fig. S2B).

Fraxini modulated cell-signaling proteins, especially c-Myc, in Hep3B cells. In addition to inhibiting anti-apoptotic proteins and increasing pro-apoptotic protein expression (Fig. 3A), Fraxini (5 µg/ml) also inhibited PI3K/mTOR pathway-associated proteins such as PI3Kα, p-4EPB1 (S65 and T37), and Fox; increased GSK3β; and reduced phosphorylated NFκB and NDRG1 in a concentration-dependent manner (Fig. 3B). Furthermore, RPPA analysis showed that Fraxini treatment led to remarkable suppression of c-Myc in Hep3B cells: Fraxini (5 µg/ml) down-regulated c-Myc expression by 84%, and the reduction of c-Myc was dose-dependent (Fig. 3B). Western blot results further confirmed that Fraxini notably inhibited the c-Myc expression in a concentration dependent manner in Hep3B cells, while it also decreased expression of pRb, and p4EBP in a dose-dependent manner, which further confirmed the RPPA results (Fig. 3C). Intriguingly, Fraxini treatment also reduced c-Myc expression in Hep3B tumor tissues compared to that of control mice (Fig. 3D).

### Fraxini’s antiproliferative activity mediated primarily by c-Myc protein

**Figure 4:**
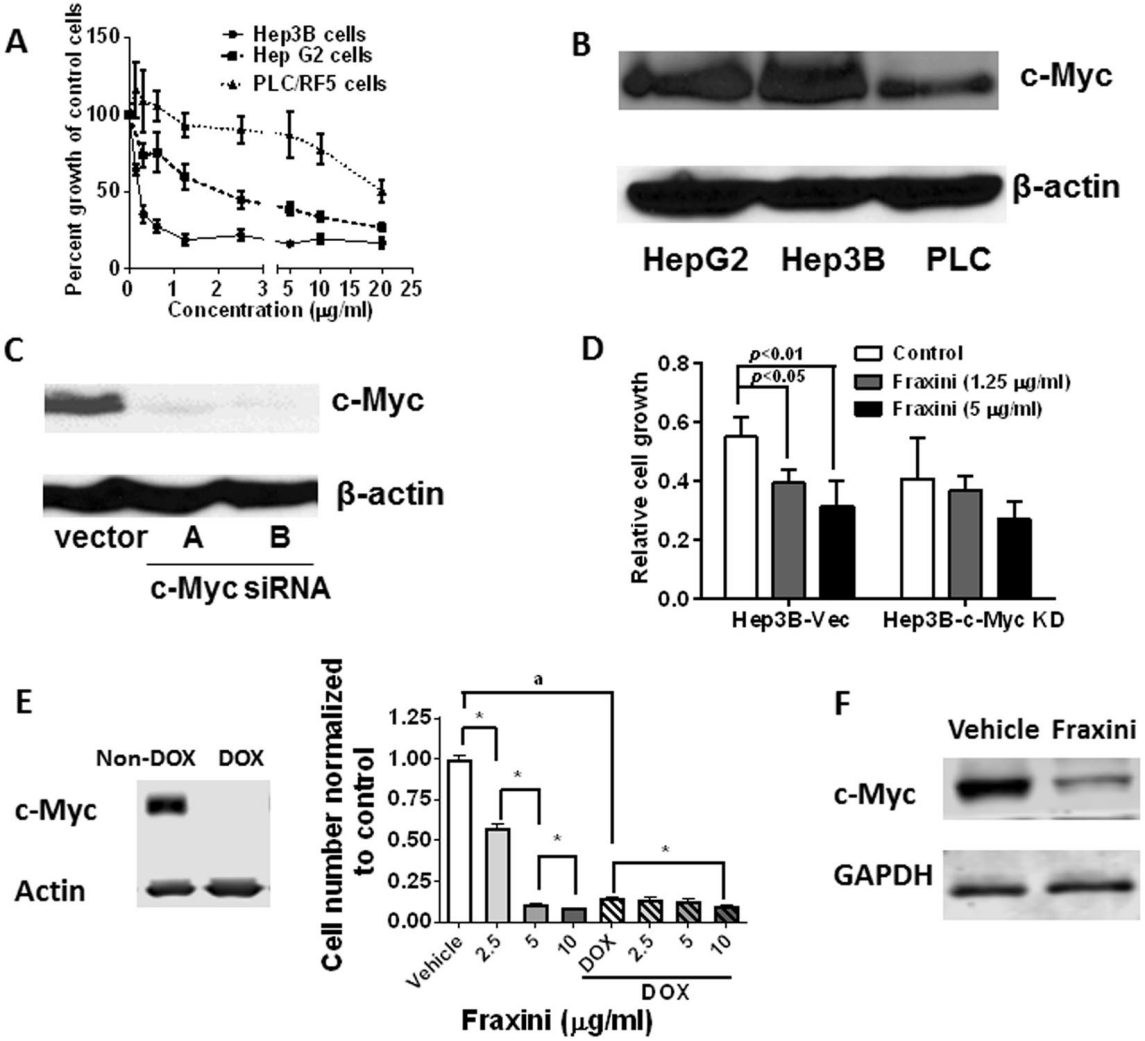
Cell growth inhibition activity of Fraxini in hepatocellular carcinoma (Hep3B and PLC/RF/5) and hepatoma HepG2 cells is dependent on c-Myc expression. (**A**) The anti-proliferative effects of Fraxini in aforementioned three liver cancer cell lines. (**B**) c-Myc levels in three liver cancer cell lines. (**C**) The expression of c-Myc in vector-transfected (Hep3B-Vec) and c-Myc siRNA–transfected Hep3B cells (Hep3B-c-Myc KD). (**D**) The proliferation of Hep3B-Vec and Hep3B-c-Myc KD cells treated with Fraxini. (**E**) Growth inhibition of Fraxini in hepatocellular carcinoma was observed in EC4 that conditionally express c-Myc and inactivate MYC by adding doxycycline (DOX, 20 ng/ml). Finally, Fraxini also reduced c-Myc protein expression in EC4 cells with c-Myc expression. (**F**) Data are presented as means ± SD. *p < 0.05 versus control vehicle treated; ^a^p < 0.05 statistical different for the vehicle treated control cells between the groups.

To further understand whether Fraxini’s down-regulation of c-Myc expression played a role in Fraxini’s anticancer activity, we compared the antiproliferative activity of Fraxini in two HCC cell lines (Hep3B and PLC) and hepatoma HepG2 cells. As shown in Fig. 4A, Fraxini was most potent in Hep3B cells (IC_50_, 0.24 µg/ml), whereas PLC cells were least responsive to Fraxini treatment (IC_50_, 20 µg/ml). Of the three cell lines, Hep3B cells had the highest c-Myc expression, whereas PLC cells had the lowest c-Myc expression, suggesting the importance of c-Myc in Fraxini’s anticancer activity (Fig. 4B). Furthermore, anti-proliferation induced by Fraxini (1.25 μg/ml) was less pronounced in c-Myc knockdown Hep3B cells (10% inhibition) than in control siRNA–transfected cells (29% inhibition) (Fig. 4C,D). Additionally, we investigated the anti-proliferative effect of Fraxini in mouse primary liver cancer cell line (EC4 cells) that over-expresses c-Myc protein which can be switched off with Tetracycline (or Doxycycline). When c-Myc protein is turned off (with doxycycline treatment), the cells are not responding to Fraxini treatment in terms of inhibition of cell proliferation compared to the cells with c-Myc protein expressed (without doxycycline treatment) (Fig. 4E). Fraxini also reduced c-Myc expression in c-Myc expressing EC4 cells (Fig. 4F).

### Fraxini’s posttranslational down-regulation of c-Myc expression

**Figure 5:**
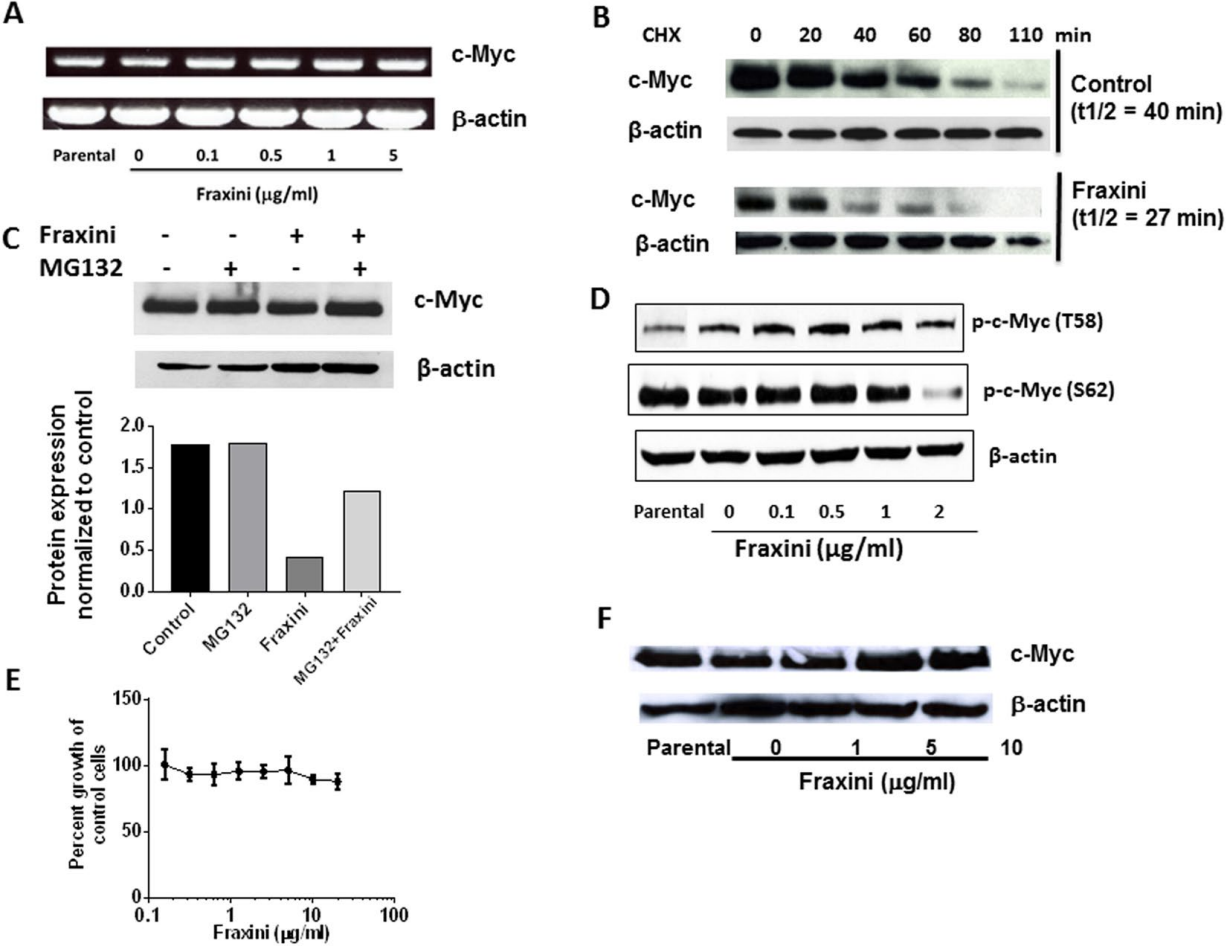
Fraxini regulated c-Myc stability in Hep3B cells. (**A**) Expression of c-Myc mRNA in Fraxini-treated Hep3B cells. (**B**) Cycloheximide (CHX) chase assay showing the half-life of c-Myc protein. (**C**) c-Myc expression in Hep3B cells treated with or without proteasome inhibitor MG-132 (400 nM). (**D**) Fraxini-regulated phosphorylation of c-Myc. (**E**) Growth curve of Fraxini-treated Burkitt lymphoma cells (Raji cells), which are known to carry T58 mutant *CMYC*. (**F**) Expression of c-Myc in Raji cells treated with Fraxini. Abbreviations: min, minute; p-, phosphorylated; T1/2, half-life of c-Myc.

We investigated whether Fraxini altered *CMYC* gene expression to reduce c-Myc protein level in Hep3B cells. Surprisingly, *CMYC* gene expression was not altered by Fraxini treatment (Fig. 5A), suggesting that the effect of Fraxini on c-Myc is mediated at the translational level rather than the transcriptional level.

Studies have shown that c-Myc is degraded through the proteasomal ubiquitination with a very short half-life. Our cycloheximide chase assay results showed that the half-life of c-Myc in Fraxini-treated Hep3B cells was lower than in vehicle-treated cells (Fig. 5B). Pretreating the Hep3B cells with MG-132, an inhibitor to reduce 26S proteasome activity for 4 hr prior to treating the cells with Fraxini, we found that Fraxini failed to reduce c-Myc levels compared with cells treated with Fraxini alone (Fig. 5C).

Because a previous study showed that the phosphorylation of c-Myc at T58 and S62 plays a pivotal role in c-Myc activity, stability, and degradation^36^, we investigated the effect of Fraxini on the phosphorylation of c-Myc. As shown in Fig. 5D, Fraxini decreased phosphorylation of c-Myc at S62 and slightly increased phosphorylation of c-Myc at T58. To confirm that Fraxini’s anticancer activity was mediated by posttranslational regulation of c-Myc, especially via regulating c-Myc phosphorylation, we tested the antiproliferative effect of Fraxini in Burkitt lymphoma Raji cells, which harbor *CMYC* T58 mutation, resulting in c-Myc stabilization^37^. Strikingly, Fraxini (up to 20 µg/ml) exerted minimal antiproliferative activity in Raji cells (Fig. 5E), which correlates with the lack of down-regulation of c-Myc expression (Fig. 5F).

### MLs and Fraxini-elicited anti-proliferative activity and down-regulation of c-Myc expression

**Figure 6:**
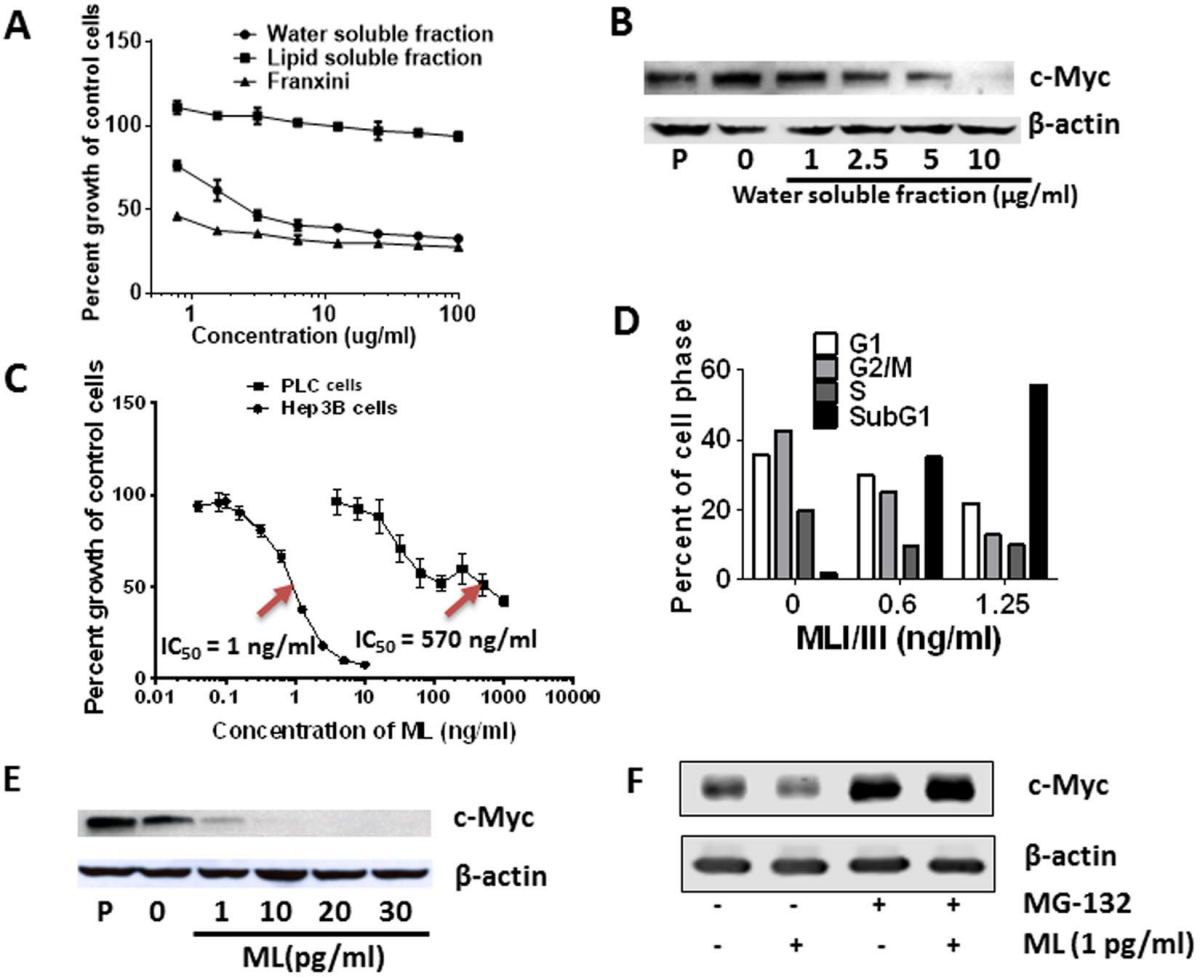
Mistletoe lectin (ML) regulated hepatocellular carcinoma cell growth and c-Myc expression. Water soluble fraction of Fraxini reduced growth of Hep3B cells (**A**) and protein expression of c-Myc (**B**). (**C**,**D**) ML was more potent in reducing the growth of Hep3B than PLC cells potentially through induction of apoptosis. ML treated Hep3B cells showed concentration dependently less expression of c-Myc protein (**E**), which was blocked by MG-132. (**F**) Abbreviations: (**P)**, parental.

To identify potential compounds responsible for Fraxini-elicited anticancer activity in HCC, we investigated the effect of water-soluble and lipid-soluble fractions of Fraxini on the growth of Hep3B cells. Proliferation of Hep3B cells was inhibited by the water-soluble fraction of Fraxini, which was similar to the anti-proliferative effects of Fraxini, but the lipid-soluble fraction of Fraxini showed minimum anti-proliferative activity in these cells (Fig. 6A). The water-soluble fraction of Fraxini also induced down-regulation of c-Myc protein expression (Fig. 6B). Further fractionation of the water-soluble components of Fraxini revealed that fraction 7 was enriched in mistletoe lectins (MLs) analyzed by the proteomic core at MDACC (Tab. S1), and was the most effective at inhibiting the proliferation of Hep3B cells with IC_50_ ≤ 1 ng/ml ML compared with the other fractions (Supporting Information Fig. S3). This finding suggests that MLs could be the bioactive components responsible for Fraxini’s anticancer activity in HCC cells.

Because MLs have anticancer and immunomodulation effects^38,39^, we treated Hep3B and PLC cells with MLs and observed that MLs reduced cell growth in both cell types in a dose-dependent manner. Intriguingly, MLs exerted much stronger anti-proliferative activity in Hep3B cells (IC_50_ < 1 ng/ml) than in PLC cells; a 6 times higher concentration of ML was required to achieve similar activity in PLC cells (Fig. 6C). Similarly, ML treated Hep3B cells underwent apoptotic cell death in a concentration dependent manner (Fig. 6D). Strikingly, a ML concentration as low as 1 pg/mL dramatically decreased c-Myc expression in Hep3B cells, and this down-regulation of c-Myc expression was dose-dependent (Fig. 6E). Additionally, we also observed that proteasome inhibition prevented reduction of c-Myc protein induced by 1 pg/ml ML (Fig. 6F). Together, all these data augment that MLs are indeed responsible for Fraxini’s antitumor activity in HCC cells.

## Discussion

We demonstrated that MEs had antiproliferative activity in HCC cell lines; of all the MEs studied, Fraxini has the most potent antiproliferative activity. Fraxini’s antiproliferative activity in Hep3B cells was associated with inducing apoptosis and mitochondrial conformational changes. Our xenograft mouse model results revealed that Fraxini suppressed Hep3B tumor development *in vivo*. By analyzing the proteomic profile of Fraxini-treated Hep3B cells, we found that Fraxini had profoundly inhibitory effect on c-Myc protein expression. We also found that MLs exerted stronger antiproliferative activity and down-regulated c-Myc expression in Hep3B cells. Given that c-Myc is crucial for HCC development and progression regardless of the etiology of disease and that MLs already have been tested in clinical trials for various tumors, our findings suggest that Fraxini could be a potentially efficacious treatment for patients with advanced HCC or with overexpression of *CMYC gene*. Further studies are needed to determine the effects of Fraxini in patients with HCC. To our knowledge, we are the first to identify the Fraxini’s anticancer activity could potentially be mediated through down-regulation of c-Myc pathway.

**Figure 7:**
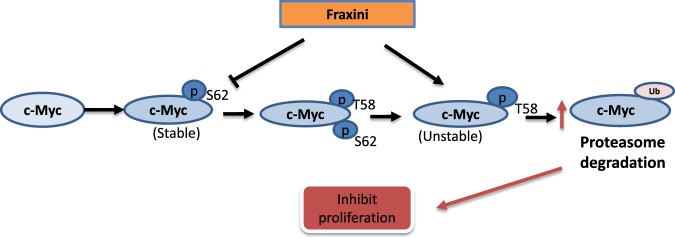
Potential mechanism associated with Fraxini elicited anti-tumor activity.

Despite long-term use of ME in cancer treatment, the mechanism of ME’s anticancer activity remains elusive. Previous studies have reported some mechanisms of the anticancer activities of MEs that primarily focus on ME’s role in immunomodulation. For example, several studies have shown that aqueous MEs can activate monocytes/macrophages, granulocytes, natural killer cells, and helper T cells and can induce various cytokines^40–44^. *Viscum album L* extract exerts cytotoxic and growth-inhibiting effects on various human tumor cell lines, lymphocytes, and fibroblasts *in vitro* by inducing apoptosis or necrosis^45,46^. Similarly, we also found that Fraxini suppressed the proliferation of Hep3B cells by inducing apoptosis and mitochondrial conformational changes. As we have also observed more vacuoles in the cell nucleus with Fraxini treatment compared to that of the vehicle treated cells, which may suggest Fraxini could also inhibit the proliferation through induction of autophagic cell death. However the examination of the expression of LC3 protein, which is a hallmark of autophagic cell death did not show increase of LC3 protein in Hep3B cells after being treated with Fraxini (Data not shown). The inhibitory effect of Fraxini on cell growth in Hep3B cells was primarily mediated through induction of apoptosis in human HCC cells. Further mechanistic study using proteomic analysis demonstrated that Fraxini could potentially affect a number of cancer cell survival and signaling pathways as illustrated in Figs 3A,B and 7 with c-Myc down-regulation being the most effective in Hep3B cells, which has not been reported previously.

*CMYC* is one of the most commonly activated oncogenes in the development of human liver cancer^47^ and is involved in diverse biologic processes, such as cell growth, apoptosis, and metabolism. Tumorigenic transcriptional factors such as KRAS, AKT, PTEN, and p53, which play key roles in cell proliferation and apoptosis, are regulated by c-Myc^48^. In mice, overexpression of *CMYC* can induce HCC, whereas inhibition of *CMYC* expression results in regression of liver tumors through the dedifferentiation of mature hepatocytes that give rise to tumors^49^. Given the potent effect of c-Myc on tumor development, it is not surprising that the cellular expression of c-Myc is regulated transcriptionally (initiation and elongation), post-transcriptionally (mRNA stability and translation), and posttranslationally (protein stability)^50^. Furthermore, in the N-terminus of c-Myc, two phosphorylation sites (T58 and S62) are critical in c-Myc protein stability. Sears *et al*. reported the phosphorylation mechanisms of T58 and S62 by Ras-activated signaling pathways ERK and GSK3β^51^. Our data indicate that Fraxini reduced c-Myc protein but not mRNA expression and slowed Hep3B tumor growth in mice. It appears that Fraxini suppressed c-Myc by decreasing c-Myc stability, which may involve the phosphorylation of both T58 and S62 on c-Myc (Fig. 7). We speculate that Fraxini regulates c-Myc stability in Hep3B cells; however, the exact mechanism by which Fraxini reduces c-Myc activity needs to be further investigated.

ML, especially MLI, has been recognized as a major bioactive component responsible for ME’s anticancer activity in various types of cancer cells^45,52,53^. In fact, several studies have shown that Aviscumine, a human recombinant MLI, induces significant cytotoxicity in various types of cancer cells by inducing either apoptosis or necrosis^54–56^. One study showed that Aviscumine activated the MAP kinase pathway, p38, Erk1/2, and SAP/JNK in ovarian cancer SKOV3 cells; activated caspases and two MAP kinase pathways in keratinocytes *in vitro*; and inhibited protein synthesis by inactivating the 60 S subunit of ribosomes^39^. In our study, ML strongly inhibited the proliferation of Hep3B cells (which had the highest c-Myc expression of the cell lines studied) but had moderate effect on PLC cells (which had the lowest c-Myc levels). These results were similar to those of Fraxini-treated cells, suggesting that the antitumor activity of both Fraxini and MLs are very likely mediated by c-Myc. Indeed, we also observed that MLs at a very low concentration (1 pg/ml) notably down-regulated c-Myc expression in Hep3B cells, which further supports the hypothesis that MLs are the bioactive components responsible for Fraxini’s antiproliferative and proapoptotic activity. Whether other bioactive components in Fraxini could contribute to Fraxini’s anticancer activity remains to be determined.

Mistletoe extracts has been studied in various clinical trials in different counties. However, across these studies, not only did the mistletoe extracts have different pharmaceutical manufacturing processes and formulations, but also the route of delivery and doses varied considerably^57^. In these reports, mistletoe extracts were administered subcutaneously, intravenously, or intravenously and subcutaneously combined. A Meta-analysis found a positive treatment effect, but did report several methodological limitations regarding the retrospective study design^58^. In terms of adverse effect, the subcutaneous administration showed more side effects, while oral administration of mistletoe preparations with apparent lack of adverse reactions might be a promising adjuvant alternative therapy for some cancer patients^59^. Although clinical studies of mistletoe are limited, a phase II study with HCC patients reported that about 20% of chemo-naïve advanced HCC patients had either CR or PR with limited toxicity after subcutaneous injection of Fraxini^13,60^. Our data here further supports a possible clinical benefit of Fraxini treatment in HCC patients. Therefore the efficacy of Fraxini and possibly the human recombinant ML, such as Aviscumine that has proper quality control minimizing batch to batch variation, in HCC patients warrants further investigation.

We showed for the first time that Fraxini potently inhibits the proliferation of HCC cells and of tumor tissue in a xenograft mouse model primarily by destabilizing c-Myc. Further research is necessary to determine whether patients with *CMYC-*overexpressing HCC show better responses to Fraxini treatment than other HCC patients without overexpression of *CMYC*.

## Methods

### Materials

All methods were performed in accordance with the relevant guidelines and regulations by The University of Texas MD Anderson Cancer Center. Iscador Q (ME grown on oak trees) and Iscador M (ME grown on maple trees) were purchased from Weleda (Arlesheim, Switzerland), and Fraxini (20 mg/ml) was purchased from ABNOBA GmbH (Pforzheim, Germany). Anti-c-Myc, anti-phospho-Rb (pRb, S807/811), anti-phospho-4EBP (p4EBP, T37/46), phospho-c-Myc (threonine 58, T58), and phospho-c-Myc (serine 62, S62) were obtained from Cell Signaling Technology (Danvers, MA). MLs, beta-actin (β-actin), and all other reagents were purchased from Sigma-Aldrich (St. Louis, MO).

### Cell culture

Hep3B, Hep G2, and PLC/PRF/5 (PLC) cells were obtained from the American Type Culture Collection (ATCC, Manassas, VA) and were maintained in a humidified atmosphere containing 5% carbon dioxide at 37 °C. Hep3B and HepG2 cells were routinely cultured in Dulbecco modified Eagle medium/F12. PLC cells were cultured in minimum essential medium. All media were supplemented with 10% heat-inactivated fetal bovine serum (HyClone Laboratories, Inc., Logan, UT), penicillin (10 IU/ml, Invitrogen/Thermo Fisher Scientific, Carlsbad, CA), and streptomycin (10 µg/ml, Invitrogen/Thermo Fisher Scientific). Human Burkitts B-cell lymphoma Raji cells were also purchased from ATCC (Manassas, VA) and were cultured in RPMI 1640 medium supplemented with 10% fetal bovine serum (FBS; Hyclone, Logan, UT), 1 mM L-glutamine, and 50 IU/ml penicillin and 50 μg/ml streptomycin. Mouse primary HCC cells (EC4) was a gift from Dr. Dean W. Felsher at Stanford University. The cells were maintain in DMEM culture medium supplemented with 10% heat-inactivated fetal bovine serum (HyClone Laboratories, Inc., Logan, UT), L-glutamine (2 mM, Gibco, Grand Island, New York), sodium pyruvate (1 mM, Gibco, Grand Island, New York), MEM nonessential amino acids (1%, Gibco, Grand Island, New York) and Anti Anti (1% antibiotics: 10,000 units/mL of penicillin, 10,000 μg/mL of streptomycin, and 25 μg/mL of Amphotericin B from Gibco, Grand Island, New York).

### Cell proliferation

HCC cells (1 × 10^4^) were plated in 96-well plates. After incubating for 24 hr, cells were treated with various concentrations of Iscador Q or M or Fraxini (0.1 µg/ml to 100 µg/ml) or Mistletoe lectins (MLs) (0.01–10 ng/ml). After an additional 72 hr, inhibition of cellular proliferation was assessed by MTT assay^61^. Absorbance was read at a wavelength of 570 nm and a reference wavelength of 650 nm using a VMax Microplate Reader (Molecular Devices, Inc., Sunnyvale, CA). The effect of Fraxini on the growth of Raji cells was assessed by the PrestoBlue assay^62^.

### Animal study

All animal experiments were approved by The University of Texas MD Anderson Cancer Center Animal Care and Use Committee. Six-week-old female BALB/c athymic (Nu/Nu) mice were purchased from the Department of Experimental Radiation Oncology at MD Anderson Cancer Center and were acclimated to the institutional animal care facility for 1 week. Mice were injected subcutaneously with Hep3B cells (5 × 10^6^ per mouse) and were placed in one of three treatment groups (n = 5 for each group): control or Fraxini (4 mg/kg or 8 mg/kg). Each group was treated via subcutaneous injection twice per week starting the day after tumor cell inoculation. Tumor take rate was 100%. Tumor growth was then measured for 3 weeks. The length and width of the tumors (mm) were measured twice per week using calipers. The tumor volume was calculated using the formula (l × w^2^) × 0.5, where l and w represent the length and the width, respectively.

### Cell-cycle and apoptosis analysis

For cell-cycle analysis, Hep3B cells (2.5 × 10^6^) grown in 100-mm dishes were treated with Fraxini (1 µg/ml–20 µg/ml) for 24 hr. Cells were subjected to trypsinization and centrifugation, and the pellets were suspended and washed in 1X phosphate-buffered saline solution (PBS) and were fixed overnight in 70% ethanol at 4 °C. The cells were then washed with 1X PBS and were suspended in PBTB staining solution containing PBS, 0.5% bovine serum albumin, 0.005% Tween-20, propidium iodide (10 μg/ml), and DNase-free RNase (1 μg/ml). Cells were incubated in the dark for 30 min at 37 °C before fluorescence-activated cell-sorting analysis (FACS) using a BD FACSCaliber flow cytometer (BD Biosciences, San Jose, CA). The percentage of cells in each phase of the cell cycle was estimated from the DNA histogram content.

The apoptotic cell death was further measured by Annexin V surface staining in Hep3B cells treated with Fraxini for 48 hr. Briefly, cells (2.5 × 10^6^) were double-stained with fluorescein isothiocyanate–conjugated Annexin V and propidium iodide per the manufacturer’s instructions (BD Biosciences). Fluorescence was detected by a BD FACSCalibur flow cytometer and was analyzed using CellQuest^TM^ Pro software (BD Biosciences).

### Transmission electron microscopy

We investigated whether Fraxini could alter intracellular structures in Hep3B cells. Cellular structure was examined by transmission electron microscopy (TEM). Hep3B cells were grown on glass coverslips for 24 hr before treatment with Fraxini (10 µg/ml). After incubating for 24 hr, cells were then washed with cold PBS and were prepared for electron microscopy, as previously described^63^.

### Reverse phase protein array

Hep3B cells (1 × 10^6^) were treated with Fraxini (5 µg/ml, 10 µg/ml, or 20 µg/ml) for 24 hr and were harvested, and cell lysates were subjected to reverse phase protein array (RPPA) analysis by the Functional Proteomics Core Facility at The University of Texas MD Anderson Cancer Center. Briefly, cells were washed twice with PBS and were suspended in ice-cold lysis buffer (1% Triton X-100, HEPES [50 mM, pH 7.4], sodium chloride [150 mM], magnesium chloride [1.5 mM], EDTA [1 mM], sodium fluoride [100 mM], sodium pyrophosphate [10 mM], sodium orthovanadate [1 mM], and 10% glycerol) supplemented with proteinase inhibitors (Roche Applied Science, Madison, WI), were diluted and subject to proteomic array analysis, as previously described^64,65^.

### Quantitative reverse transcription-polymerase chain reaction

We used quantitative reverse transcription-polymerase chain reaction (RT-PCR) analysis to compare *CMYC* gene expression in Hep3B cells. Total RNA was prepared from Hep3B cells treated with Fraxini with the doses indicated in the figure for 1 day, and cDNA was generated. RT-PCR primers for *MYCC* were as previously described^66^:

Forward: 5′–TACCCTCTCAACGACAGCAG–3′

Reverse: 5′–TCTTGACATTCTCCTCGGTG–3′

Primers for β-actin were used^67^:

Forward: 5′–GATGATGATATCGCCGCGCTCGTCGTC–3′

Reverse: 5′–GTGCCTCAGGGCAGCGGACCGCTCA–3′

RT-PCR was done following the manufacturer’s protocol (Invitrogen/Thermo Fisher Scientific). Target gene expression levels were normalized to the β-actin expression level for each sample. PCR conditions for *CMYC* included an initial denaturation step (4 min at 95 °C) followed by 35 cycles of denaturation (94 °C for 30 sec), annealing (60 °C for 30 sec), annealing (72 °C for 45 sec), and a final extension step (72 °C for 5 min). The conditions for β-actin were the same.

### Immunoblotting

Hep3B cells were treated with Fraxini, water-soluble fraction of Fraxini (preparation procedure of water-soluble fraction of Fraxini was illustrated in Fig. S1), or MLs for 24 hr and then were harvested in sodium dodecyl sulfate buffer. Total protein (50 μg per well) was separated on a 10–15% sodium dodecyl sulfate gel and was transferred onto polyvinylidene fluoride membranes. The membranes were blocked with 5% nonfat milk in Tris-buffered saline solution and then were probed with primary antibodies at 4 °C overnight. The membranes were visualized using SuperSignal substrate (Pierce ECL Plus, Thermo Fisher Scientific). β-actin was detected for normalization of results.

### siRNA transfection

Hep3B cells were plated in 6-well plates and allowed to attach overnight. Transient transfection of nonspecific siRNA (control siRNA) and c-Myc siRNA (OriGene, Rockville, MD) was carried out using Lipofectamine RNAiMAX Transfection Reagent (Thermo Fisher Scientific) following the manufacturer’s instructions. Beginning 48 hr after transfection, cells were treated with Fraxini for 24 hr. Protein was collected for Western blot analysis.

### Data analysis

Data were expressed as means ± SD. To analyze differences between groups, Student *t*-tests were used to analyze mean numerical data. Data were analyzed using SPSS software (version 20; IBM Corporation, Armonk, NY). *p* levels less than 0.05 were considered statistically significant.

## Supplementary information

Figure S1
