# Supplementary material for: Mistletoe extract Fraxini inhibits the proliferation of liver cancer by down-regulating c-Myc expression
Source: Sci Rep. 2019 Apr 23;9:6428. doi: 10.1038/s41598-019-41444-2 (PMC6478697; doi:10.1038/s41598-019-41444-2)

**Mistletoe extract Fraxini inhibits the proliferation of liver cancer by down-regulating c-Myc expression**

Peiying Yang^1*^, Yan Jiang^1^, Yong Pan^1^, Xiaoping Ding^2^, Patrea Rhea^1^, Jibin Ding^1^, David H. Hawke^3^, Dean Felsher^4^, Goutham Narla^5^, Zhimin Lu^6^, and Richard T. Lee^7^

^1^Departments of Palliative, Rehabilitation, and­ Integrative Medicine, The University of Texas MD Anderson Cancer Center, Houston, Texas, USA.

^2^Hubei Institute for Food and Drug Control, Wuhan, Hubei, P.R. China.

^3^Department of System Biology, The University of Texas MD Anderson Cancer Center, Houston, Texas, USA.

^4^Department of Departments of Medicine and Pathology, Stanford University School of Medicine, San Francisco, California, USA

^5^Departments of Medicine and Genetics, University Hospitals Case Medical Center and Case Western Reserve University, Cleveland, Ohio, USA

^6^Department of Neuro-Oncology, The University of Texas MD Anderson Cancer Center, Houston, Texas, USA.

^7^Department of Medicine, University Hospitals Case Medical Center & Case Western Reserve University, Cleveland, Ohio, USA

A


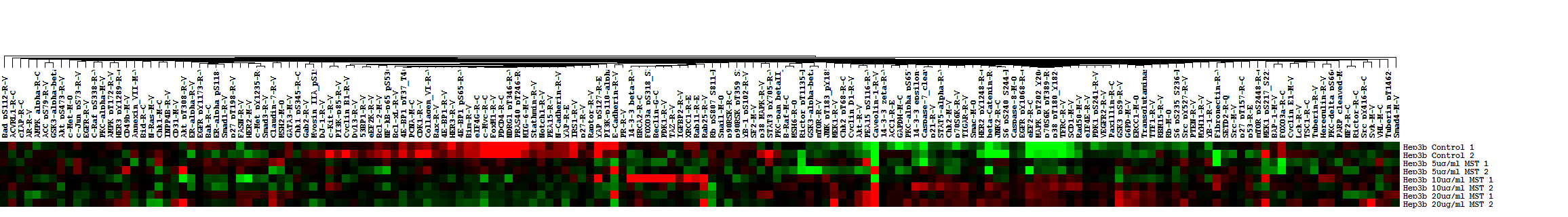


B


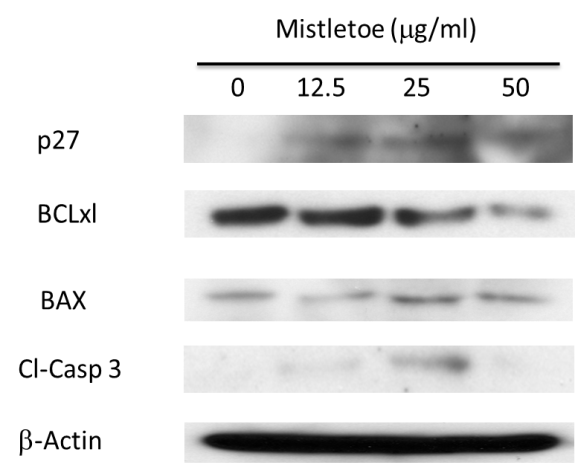


Abbreviations: Cl, cleaved; casp, caspase.

**Figure S2.** Heating map for RPPA results (A) and western blotting of the apoptotic regulating protein in Hep3B cells treated with Fraxini (B).

**Figure S3.** Effect of fractions of Fraxini on Hep3B cell proliferation after 72 hr treatment.

Table S1. Protein identification in Fraction #7 of Fraxini and mistletoe lectin

| ***Mass/Name*** | ***Fraction 7*** ***(Score/EMPAI)*** | ***Mistletoe lectin (Sigma)******(Score/EMPAI)*** |
| --- | --- | --- |
| 62589/ML1 VISAL | 1347/4.70 | 5441/10.49 |
| 62646/ML3 VISAL | 512/0.76 | 1620/2.76 |
| 24394/TRYP PIG | Etc/0.67 | 1.03 |
| 65999/K2C1 Human | 0.71 | 0.22 |
| 99425/CHO2 CANGA |  | 0.05 |
| 43704/CYDE METM7 |  | 0.1 |
| 60008/K2C6A Human | 0.31 |  |
| 69088/DNAK ECOHS | 0.26 |  |
| 48911/RBL DARCA | 0.22 |  |
| 51529/K1C14 HUMAN | 0.13 |  |
| 41980/ALFC ORYSJ | 0.16 |  |
| 58792/K1C10 HUMAN | 0.11 |  |
| 16784/FUR ECOLI | 0.20 |  |
| 31495/BLAT ECOLX | 0.11 |  |

Fractions for gel-filtration chromatography were acetone precipitated and digested with 200 ng of trypsin (sequencing grade, Promega) for 18 hrs at 37C in the presence of Rapigest (Waters). Resulting peptides were extracted and analyzed by high-sensitivity LC-MS/MS on an Orbitrap Elite or Orbitrap Fusion mass spectrometer (Thermo Scientific, Waltham MA). Proteins were identified by database searching of the fragment spectra against the SwissProt (EBI) protein database using Mascot (v 2.5, Matrix Science, London, UK). Typical search settings were: mass tolerances, 10 ppm precursor, 0.8d fragments; variable modifications, methionine sulfoxide, pyro-glutamate formation; trypsin, up to 2 missed cleavages.

**Supplement Fig S4**. Original Western blots of cell signaling proteins, c-Myc protein, β-actin protein and GAPDH protein for the revised manuscript. In each gel, we indicted the blot with the information for the proteins tested by Western blot which was presented in Figure 3-6 in the manuscript. The black line indicate the bands of the different proteins which were cropped to show in the figure 3-6. The antibodies used are listed under the Material and Method.

Cell signaling protein blots showed in Figure 3.


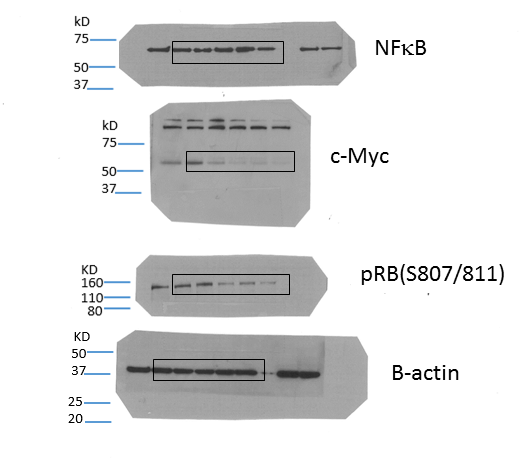


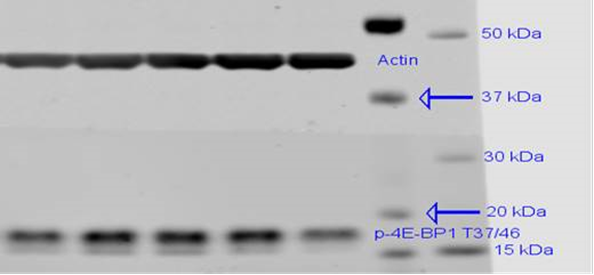


c-Myc and beta-actin blots showed in Figure 4B and 4C.


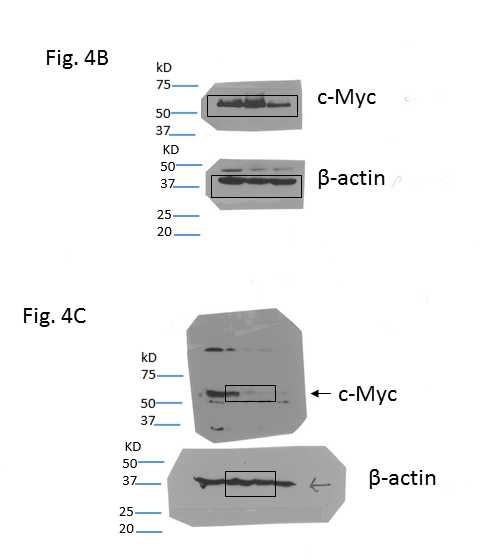


c-Myc and β-actin blots showed in Figure 4E and 4F


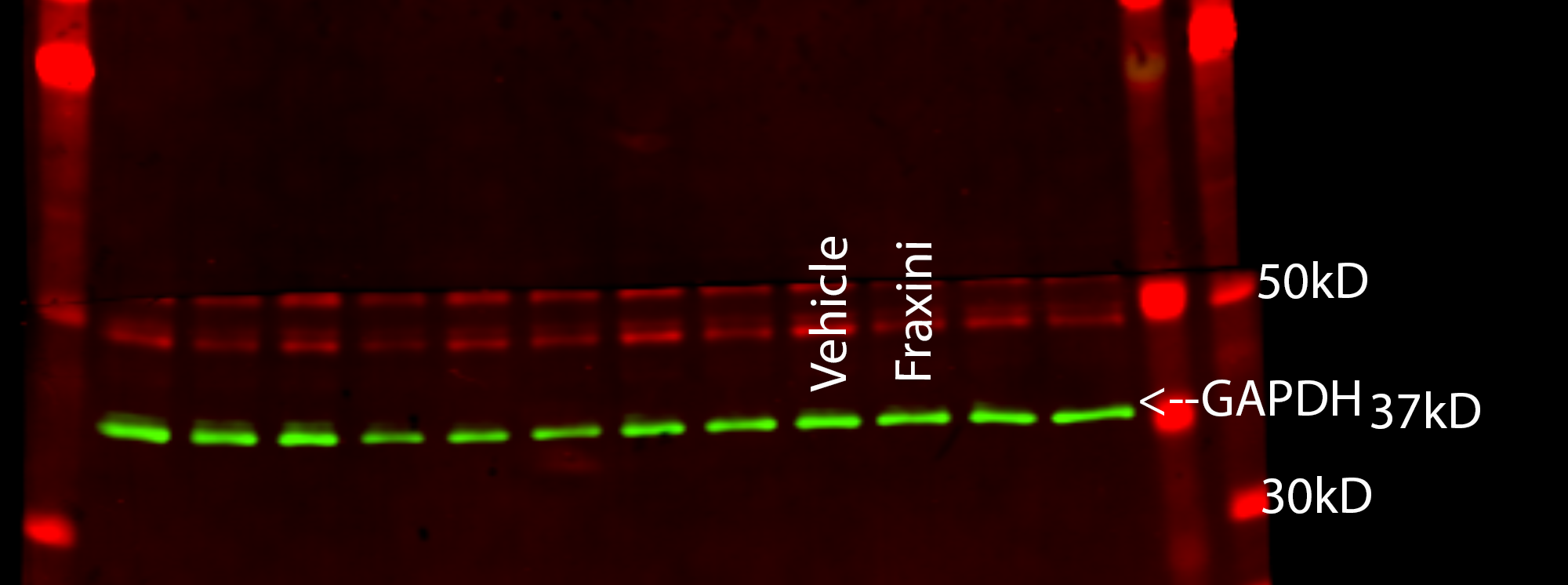

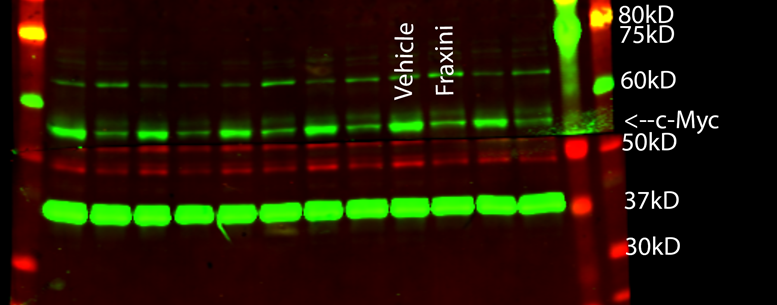

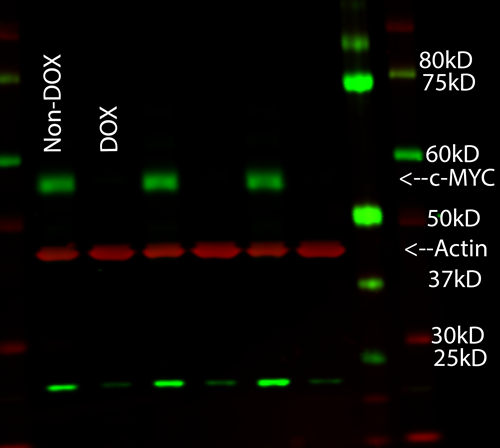


Figure 4E

Figure 4F

C-Myc and β-actin blots showed in Figure 5B and 5C


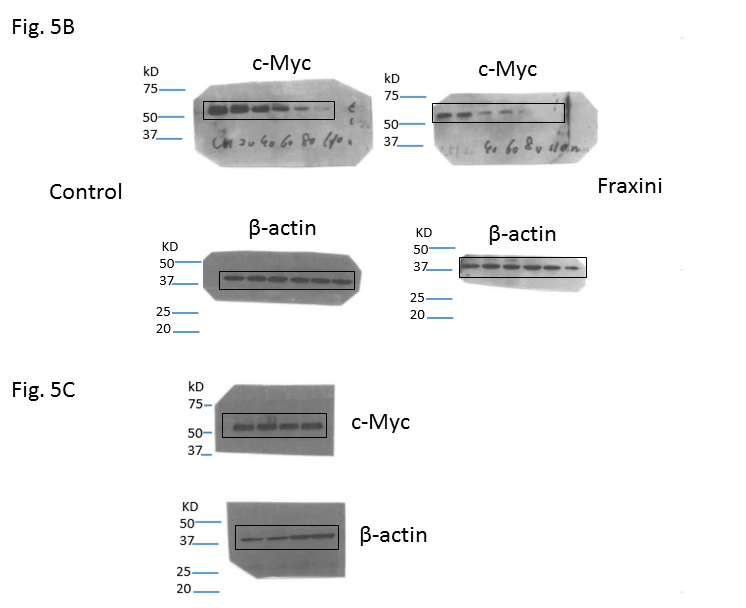


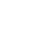
 C-Myc, p-cMyc and β-actin blots showed in Figure 5D and 5F


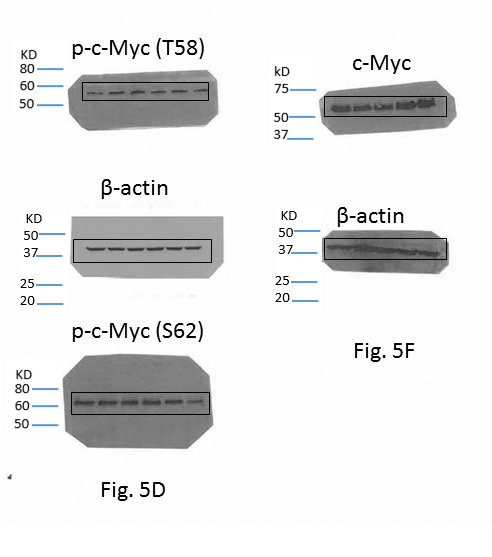


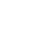


c-Myc and β-actin blots showed in Figure 6


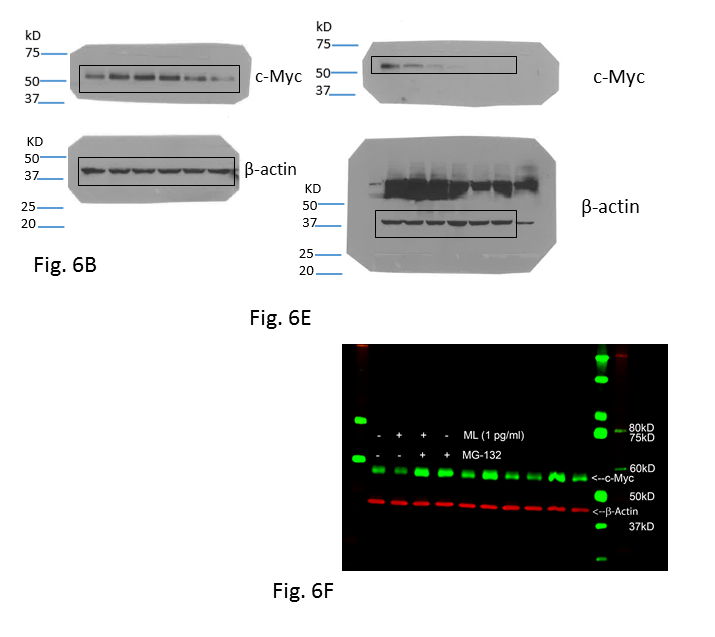

Supplement: Supplementary file 1 — Figure S1 [file 41598_2019_41444_MOESM1_ESM.doc]
